# *In vivo *study of experimental pneumococcal meningitis using magnetic resonance imaging

**DOI:** 10.1186/1471-2342-8-1

**Published:** 2008-01-14

**Authors:** Christian T Brandt, Helle Simonsen, Matthew Liptrot, Lise V Søgaard, Jens D Lundgren, Christian Østergaard, Niels Frimodt-Møller, Ian J Rowland

**Affiliations:** 1 National Center for Antimicrobials and Infection Control, Statens Serum Institut, Copenhagen, Denmark; 2 Danish Research Centre for Magnetic Resonance, Copenhagen University Hospital Hvidovre, Copenhagen, Denmark; 3 CHIP, Faculty of Health Sciences, University of Copenhagen, Denmark; 4 Clinical Microbiological Department, University Hospital Herlev, Copenhagen, Denmark; 5 Radiology, University Wisconsin-Madison, Clinical Science Center, 600 Highland Avenue, Madison, WI 53792, USA

## Abstract

**Background:**

Magnetic Resonance Imaging (MRI) methods were evaluated as a tool for the study of experimental meningitis. The identification and characterisation of pathophysiological parameters that vary during the course of the disease could be used as markers for future studies of new treatment strategies.

**Methods:**

Rats infected intracisternally with *S. pneumoniae *(n = 29) or saline (n = 13) were randomized for imaging at 6, 12, 24, 30, 36, 42 or 48 hours after infection. T1W, T2W, quantitative diffusion, and post contrast T1W images were acquired at 4.7 T. Dynamic MRI (dMRI) was used to evaluate blood-brain-barrier (BBB) permeability and to obtain a measure of cerebral and muscle perfusion. Clinical- and motor scores, bacterial counts in CSF and blood, and WBC counts in CSF were measured.

**Results:**

MR images and dMRI revealed the development of a highly significant increase in BBB permeability (P < 0.002) and ventricle size (P < 0.0001) among infected rats. Clinical disease severity was closely related to ventricle expansion (P = 0.024).

Changes in brain water distribution, assessed by ADC, and categorization of brain 'perfusion' by cortex ΔSI_(bolus) _were subject to increased inter-rat variation as the disease progressed, but without overall differences compared to uninfected rats (P > 0.05). Areas of well-'perfused' muscle decreased with the progression of infection indicative of septicaemia (P = 0.05).

**Conclusion:**

The evolution of bacterial meningitis was successfully followed *in-vivo *with MRI. Increasing BBB-breakdown and ventricle size was observed in rats with meningitis whereas changes in brain water distribution were heterogeneous. MRI will be a valuable technique for future studies aiming at evaluating or optimizing adjunctive treatments

## Background

The development of bacterial meningitis is associated with multiple pathophysiological changes in brain homeostasis. Previously, this has been investigated experimentally using different methodologies to determine the kinetics of infection and inflammation; loss of blood-brain-barrier and blood-labyrinth-barrier; damage to brain cortex, hippocampus and white matter; development of brain oedema; alterations in brain blood supply and loss of cerebral vascular autoregulation [1-6]. However, experimental investigations of the interaction between these pathophysiological measures have been limited, probably due to complexity and incompatibility of the methodologies applied. Recent bioluminescence studies illustrate the apparent limitation of a single method, where the optical technique is able to visualize the dynamics of the progressing meningeal infection but does not provide information relating to disease induced physiological changes [7,8].

Whilst magnetic resonance imaging (MRI) has been widely used for the study of experimental stroke [9], its application has been limited in studies of experimental meningitis [10-12], with reported studies primarily focusing on visualization of meningeal enhancement and hydrocephalus at low resolution. When compared to experimental stroke studies that aim to produce single lesions localised in predetermined anatomical sites, bacterial meningitis results in diffuse and unpredictable involvement of brain vasculature and parenchyma that is often complicated by systemic infection [13].

In clinical neuroinfections, including meningitis, MRI has primarily been used as a diagnostic tool to assess brain pathology, intracranial complications and to evaluate responsiveness to treatment [14,15], despite MRI's capability to provide quantifiable *in vivo *data on BBB function, brain water distribution as well as indices of cerebral blood supply [9].

Consequently, MRI methodology was used to acquire multi-parametric *in-vivo *data to characterize temporal changes during the course of experimental pneumococcal meningitis in the rat model. MR measures of inflammation, vascular permeability, brain water, brain contrast agent supply and pathoanatomy were obtained together with clinical and paraclinical data, in both infected and control animals. Identification of parameters that report on the disease progress may be used in future studies to assess and optimize therapeutic strategies.

## Methods

The experimental protocol was approved by the Danish Animal Inspectorate (Dyreforsoegstilsynet). Adult male Wistar rats (280–300 g) were used for the experiments. Normal day/night cycles and free access to food and water were provided.

### Experimental study design

Data from 29 rats infected with pneumococci and 13 controls inoculated with saline are presented. Four infected and 2 control rats were randomized to each MR examination time point at 6, 12, 24, 30, 36, 42 or 48 hours following inoculation. Immediately prior to MR investigation, each rat was assessed clinically and neurologically (see Table 1). After imaging, blood and CSF samples were taken and brains prepared for histopathology (see below). One infected and 1 control rat, assigned to MR imaging at 36 and 48 hours after inoculation, died in the scanner prior to image acquisition. One infected rat (24 hours) died immediately prior to contrast administration. Also, contrast administration was not successful in two rats assigned to MR imaging at 6 and 42 hours after infection. The two dead infected rats were replaced from a separate group that was not assigned to a specific time point *a priori *(the group was incorporated due to the risk of sick rats dying from anesthesia, n = 4. Spare controls were not incorporated). Therefore, datasets from 5 infected rats were included for the 24 hour time point, providing a total of 29 datasets, whereas contrast administration data was only obtained in 26 infected rats.

**Table 1:** Guidelines for clinical disease score and motorical performance score.

**Motor Function**				
**Grade**	**Movement**	**Front legs** *(Held by the tail, normally the front paws grip the edge of i.e. a table)*	**Hindlegs** *(Hind legs are placed below a table edge. Normal reaction is an immediate grip of the edge).*	**Climbing** *(Plate with an upward slope of 30 degrees. The rat will normally crawl upwards and grip the top edge).*

**0**	Normal.	Both legs grip the table edge and is cabaple of "walking along the edge".	Normal.	Normal.
**1**	Movements slower. Can still be activated.	Both legs grips the edge. No movement.	Seeking to grip the table edge.	Does nor crawl upwards but spreads legs to hold on.
**2**	Prefers to stay in the same place. Nose is still active. Still climbing, Paws are seeking.	Only one leg grips the edge or no gripping.	Does not grip the edge.	Slides down.
**3**	Developing problems with controlling the limbs. Does not move. Tilting. Front paws sliding.			
**4**	Paresis of one or more extremities.			

**Clinical Appearance**				

**Grade**	**Activity**	**Eyes**	**Fur**	

**0**	Normal activity.	Normal eyes and surroundings.	Tended fur.	
**1**	Activity reduced, Turns easily if laid on the back.	Wet eyes.	Pilo-erection.	
**2**	Slow turning if laid on the back.	Haemorrhage around eyes. Scrathcing of eyes.	Pilo-erection and untended fur.	
**3**	Does not turn around.			
**4**	Lying on the side.			

### Infection

A *Streptococcus pneumoniae *type 3 strain (68034, Statens Serum Institut (SSI), Copenhagen, Denmark) was used for the experiments [4]. The infectious inoculum was diluted in cold saline to a final concentration of 2–5 × 10^5 ^CFU/ml, as confirmed by quantitative cultures. On the day of inoculation, rats were anaesthetized subcutaneously with 3 ml/kg weight of an aqueous solution (10 ml sterile water) of Hypnorm^© ^(2 ml)/Dormicum^© ^(4 ml, 5 mg/ml)/Atropine (1.5 ml, 1 mg/ml) and injected intracisternally with 30 μl of the bacterial suspension or saline using a butterfly syringe. After MR investigation, cerebrospinal fluid (CSF) and blood was obtained by cisterna magna- and tail-vein puncture from each animal. White blood cell (WBC) counts in CSF were measured on an automatic cell counter (Swelab Autocounter AC 920, Swelab Instruments, Sweden) using 20 μl of CSF. Bacterial counts in CSF were determined by plating 10-fold serial dilutions of 20 μl CSF. Fifty μl of undiluted blood and 50 μl of a 20-fold dilution were plated. CSF from uninfected controls was plated undiluted.

### Assessment of clinical disease, motor performance and disease severity (Table 1)

Clinical appearance and motor-function scores were determined prior to each scanning session. The scoring method was based on previous reports and fitted to the present model of disease [1,16,17], see Table 1. Overlap between scores was evident since, for example, ambulatory activity would be affected by clinical disease.

### MR imaging

MR imaging was performed using a Varian SISCO 4.7 Tesla imaging and spectroscopy system. Rats were positioned in a stereotactic device placed within a home-built quadrature coil. The animals were kept warm using a thermostatically controlled blanket with circulating warm water (35°C). All rats underwent T1-weighted (T1W), T2-weighted (T2W), quantitative diffusion, dynamic MRI (dMRI) and post contrast T1W measurements whilst anaesthetized as described above. To enable direct comparison of the imaging data, the same 12 contiguous coronal slices were acquired for the T1W, T2W and diffusion measurements. The acquisitions using a b-value = 0 provided the T2W images.

**Figure 1:**
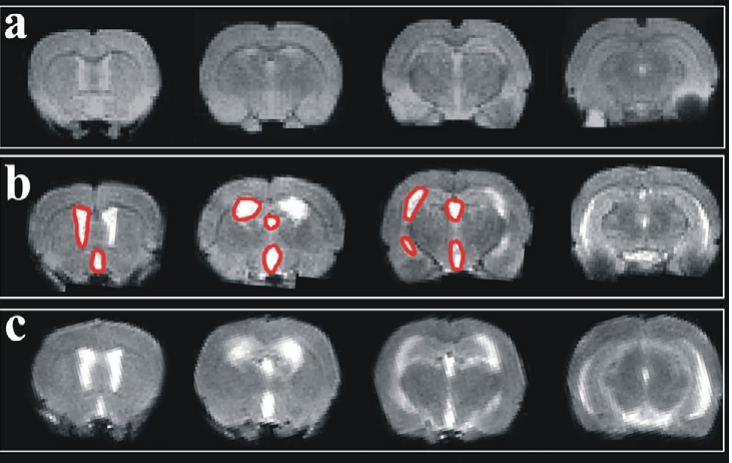
**Hydrocephalus in experimental meningitis**. T2W images showing a control rat (a) and two infected rats imaged at 36 hours (b) and 42 hours (c) presenting significant dilation of lateral and third ventricles (outlined in red) indicative of hydrocephalus. Brain-ventricle ratio (VBR) in a, b and c was 0.025, 0.076 and 0.085, respectively. The anatomical positioning of the three slices (from left to right) was used to analyze and measure VBR, ADC and dMRI.

Three coronal MR images corresponding to the frontal, mid-frontal and mid-brain (sections shown in Fig. 1) were selected for dynamic MRI investigation, measurement of ventricular-brain ratio (VBR) and calculation of ADC.

### ADC – Apparent diffusion coefficient mapping

Quantitative diffusion measurements (single in-plane direction, along the x-axis) were performed before the administration of contrast agent (echo time (TE) = 80 ms, repetition time (TR) = 2000 ms, matrix size (MA) = 128 × 128, field of view (FOV) = 40 × 40 mm, number of transients (NT) = 1 with b-values of 0, 185, 740, 1665 s/mm^2^). Regions of interest (ROI) were drawn by hand around cerebral neo-cortex, excluding the meninges. A circular ROI was used in the basal ganglia. ROI's were drawn independently by two of the authors (HS and CTB) blinded to all other data. ROI's were drawn and mean ADC's calculated using MATLAB©.

### Dynamic MRI – Loss of blood-brain-barrier (BBB) integrity and contrast agent bolus induced changes in signal intensity (ΔSI_(bolus)_) in cerebral cortex and muscle (Fig. 2)

**Figure 2:**
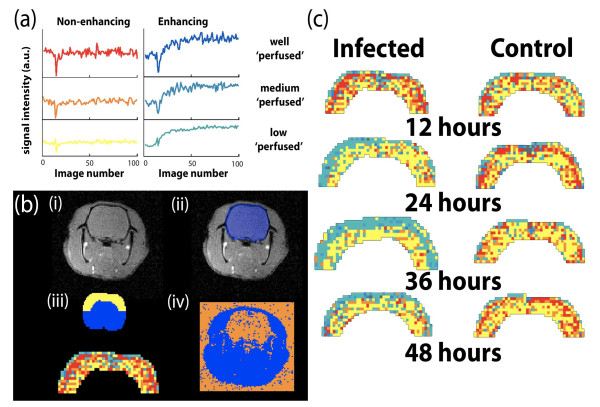
**Dynamic MRI data analysis. (a) Categorization of dMRI profiles**: Typical data sets showing dynamic MRI signal intensity profiles categorized into non-enhancing and enhancing parent classes and high, medium and low ΔSI_(bolus) _sub classes. Enhancing and non-enhancing pixels were identified according to whether curves returned to baseline (non-enhancing) or increased (enhancing) above baseline after the signal intensity reached its minimum value due to T2* losses. The extent of T2* induced signal loss determined the pixel sub-class and was, subsequently, assigned a colour. **(b) Automatic cortex region of interest selection: **Proton image (i) also shown with a brain mask (ii) calculated using thresholding and morphology. Morphological erosion of the brain mask (blue) yielded a cortex mask (iii) enabled an automatic, unbiased selection of a cortex region of interest (yellow). In addition, maps of the parent classes were obtained as shown in (iv) showing pixels that underwent contrast enhancement (blue) and those that failed to enhance (orange). **(c) Cortical regions of interest: **Cortex brain masks show the distribution of non-enhancing voxels with high (red), medium (orange) and low (yellow) ΔSI_(bolus) _values. Green voxels, dominant in outer cortex layers from rats with meningitis, show enhancing voxels indicative of blood brain barrier breakdown.

T1W images (TE = 11 ms, TR = 450 ms, SL = 1.8 mm, MA = 128 × 256, FOV = 40 mm × 40 mm, NT = 4, 12 contiguous slices) were acquired before and directly after acquisition of dMRI. Dynamic MRI data was acquired using a bolus administration of contrast agent (Magnevist, Schering AS) administered via a cannulated tail vein. The contrast agent, containing gadolinium diethylene triamine pentaacetic acid (GdDTPA), was injected within 10 seconds, at a dose of 0.5 mmol/kg. Bolus passage was followed using a dMRI protocol where 100 sets of FLASH T1W images (3 slices) were obtained with 10 images acquired before and 90 images acquired after a single injection of contrast agent (TE = 4 ms, TR = 11 ms, flip angle (FL) = 7°, SL = 1 mm, MA = 128 × 128, FOV = 40 × 40 mm, NT = 1).

#### Loss of BBB integrity – contrast agent bolus induced changes in signal intensity (ΔSI_(bolus)_) in cerebral cortex

An automated selection of cerebral cortex was performed via standard image processing methods. Thresholding and morphology were used to identify the full brain mask including ventricles, basal ganglia and the base of the brain. Morphological erosion and subsequent subtraction then extracted a region of interest, which was then halved to obtain the cortex used for analysis (see Fig. 2b). Data and results from the cortex voxels are presented as the fraction of the total number of voxels in each brain cortex selection.

A preliminary analysis of the dynamic MRI data sets using k-means clustering was performed to identify specific enhancement patterns associated with normal and infected animals. From this analysis, typical enhancement profiles were identified which could be divided into temporal regions. Consequently, data was divided into 2 major classes (non-enhancing and enhancing) and into 3 sub-classes of cortex ΔSI_(bolus) _values: 1) high, 2) medium and 3) low. Enhancing and non-enhancing voxels were classified according to whether significant T1-weighted enhancement occurred within the voxel following gadolinium administration. Sub-classification of the 3 cortex ΔSI_(bolus) _values was performed according to the extent of T2* signal loss during the bolus passage. The T2* signal loss is dependent on the concentration and distribution of gadolinium within the tissue and will, consequently, be dependent on a variety of physiological parameters including perfusion, blood volume, flow and vascular permeability and dimensions. Hence, whilst the extent of signal loss only reflects tissue perfusion, the approach does provide a semi-quantitative parameter that gives an indication of how meningitis affects brain and muscle physiology.

#### Muscle perfusion

A class of high muscle ΔSI_(bolus) _was identified in intrinsic- and extrinsic muscle in the rat head including jaw and temporal muscle. This class was not observed within the brain compartment and analysis was based on extra-cranial pixels identified by visualizing the rat head subtracting all other profiles thus clearly showing only the muscle relief of the rat head. Consequently, the analysis did not use ROI's but used extra-cranial pixels automatically assigned to the class. A reduced number of voxels in this cluster was interpreted as a reduction in muscular blood supply. Data for the infected animals are presented as a fraction of the corresponding control rat value at each time point since the study design was performed so that 2 meningitis rats were matched with one control. Failure to acquire complete data from one 48 hour control rat meant that correspondent data from 2 infected rats were unavailable.

### Measurement of ventricle-brain ratio (Fig. 1)

Regions of interest around whole brain and lateral- and third ventricles were drawn on T2W images. The ventricle-brain ratio (VBR) was calculated as ventricular area divided by total brain area for all three coronal slices included. Regions of interest (ROI's) and measurements were performed in MATLAB© and done blinded to all other data (HS).

### Histopathology

After MR investigation, rats were euthanized with pentobarbital (200 mg/ml) and perfused with 1.5% paraformaldehyde (PFA) via the left ventricle of the heart. Brains were harvested and stored for 14 days in 1.5% PFA and frozen in n-hexane mixed and cooled with dry-ice. Two 45 μm thick coronal cryosections adjacent to each other, corresponding to the MR images obtained were stained with Hematoxylin & Eosin (HE) in order to identify the nature of pathological features apparent in the MR images.

### Statistical analysis

Data are presented as mean +/- SEM. Comparisons between controls and infected rats were performed by two-way ANOVA and P < 0.05 was considered significant.

Due to the temporal development of the disease, two-way ANOVA was performed in 3 intervals for each dataset; 6 to 48 hours, 6 to 30 hours and 36 to 48 hours after infection.

Statistical comparisons between infected and control animals were only performed between MRI-generated datasets. Since muscle ΔSI_(bolus) _data were presented as a fraction of results obtained in the correspondent control, a linear regression analysis of this dataset was performed.

To identify relationships between clinical, paraclinical and MRI data, Spearman Rank correlations were performed among meningitis rats in the terminal disease phase, 36 to 48 hours after infection. Since only a limited number of comparisons were performed, a P-value below 0.05 was also considered significant for correlation analysis.

## Results

### The evolution of pneumococcal meningitis in the experimental model (Fig. 3 and 4)

**Figure 3:**
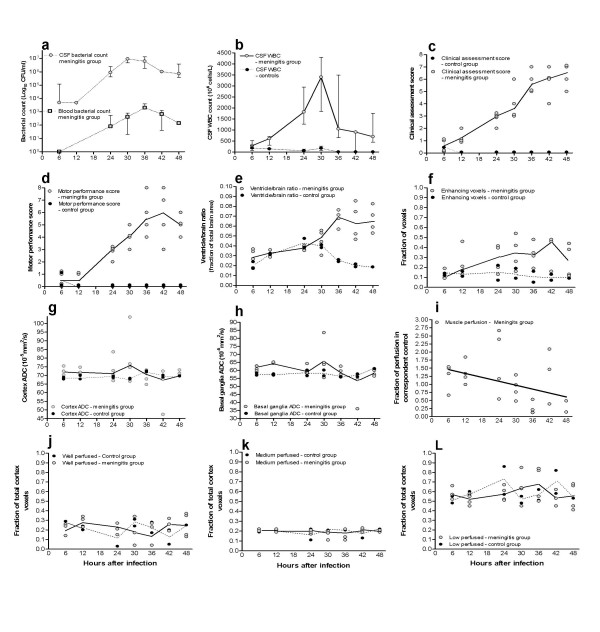
**Development of pneumococcal meningitis**. Graphs (a) to (l) show the development and changes in all included study parameters from 6 to 48 hours after inoculation in infected (n = 29, open circles and solid median line) and control rats (n = 13, black circles and dashed median line). Graph (a) and (b) show median and interquartile range of bacterial counts and WBC counts. Graph (c) and (d) show the steady worsening of clinical disease and deteriorating motor performance among infected animals. The ventricle-brain ratio (VBR) in (e) was subject to marked development among infected rats from 30 hours after infection and all infected rats had increased VBR from 36 hours onwards (P < 0.0001). The number of enhancing cortical voxels (f), indicative of BBB breakdown, was significantly increased among meningitis rats (6 to 48 hours, P = 0.0019). Graphs (g) and (h) illustrate comparable ADC values in cortex and basal ganglia among infected rats with increased variation around the mean values from the control group until 36 hours after infection (P > 0.05). Graph (i) shows decreased areas of high ΔSI_(bolus) _muscle (no. of voxels) in infected rats presented as a fraction of the value in corresponding control animals at each time point (P = 0.05). Graphs (j), (k) and (l) show the total number of voxels (enhancing + non-enhancing) in each ΔSI_(bolus) _category (P > 0.05).

**Figure 4:**
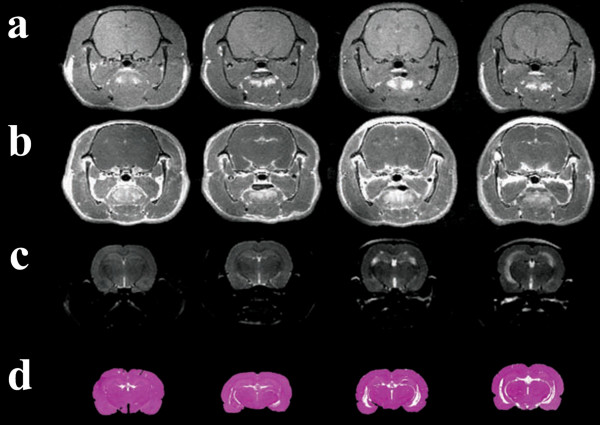
**Disease evolution visualized using MRI**. Pre- and post contrast T1W images (a, b), T2W (c) images together with equivalent Histological slices (d) illustrate the evolution of the disease. Postcontrast T1W images were used to identify meningeal enhancement. Meningeal enhancement could be visually graded as (from left to right): 0) No enhancement, 1) Thin brim of enhancement, 2) Thick brim of enhancement and 3) Diffuse enhancement with unclear borders towards outer cortex layer. T2W images (c) clearly show increasing ventricular size that was also apparent in the histological slices.

As shown in Fig. 3, the increase in the paraclinical (CSF and blood bacterial counts, CSF WBC) and MRI data (ventricle-brain ratio (VBR), Apparent Diffusion Coefficient (ADC) and dynamic MRI (dMRI)) was non-linear as the disease progressed with time. The dual phase nature of the increases suggested that this model of the disease could be characterised by a *Developing disease phase *(up to and including 30 hours after infection) followed by a *Terminal disease phase *(from 36 to 48 hours).

#### Infection and inflammation (Fig. 3a and 3b)

All CSF samples obtained from control rats were sterile. CSF and blood samples taken 6 to 48 hours after infection delineated the progression of meningitis with increasing CSF bacterial growth, secondary bacteremia and increasing CSF inflammation. Both CSF WBC and CSF bacterial counts peaked at 30 hours after infection. A plateau relieved by a downward slope in CSF WBC and CSF bacterial counts was observed in samples obtained from 30 hours onwards, declining until the final 48 hour study point. Blood bacterial counts showed that secondary bacteremia was present in one rat from 12 hours after infection and 3/4 rats at 24, 30 and 36 hours and 4/4 rats at 42 and 48 hours.

#### Development in disease scores (Fig. 3c and 3d)

As expected, clinical disease severity increased in infected animals as the disease progressed. Subsequent to increasing clinical scores, motor performance, mainly ambulatory activity, deteriorated. Increasing inter-rat variation in both clinical and motor performance scores was observed as the infection developed, being marked from 30 hours onwards. In the terminal disease phase (36 to 48 hours) no further change in the scores was observed in either clinical or motor performance scores among infected rats. Infected rats imaged at 36, 42 and 48 hours after infection were comparable with respect to clinical disease and motor performance.

Following intracisternal inoculation of saline, clinical disease was not observed among these control rats although slight post-anaesthetic drowsiness (the Animal Inspectorate required animals to be transported to the MR centre whilst anaesthetised) accounted for a score of 1 in one control at 6 hours after inoculation.

#### Ventricle-brain ratio – expansion of the lateral and third ventricle (Fig. 1, 3e and 4)

The extent of ventricle expansion, determined as the ventricle-brain ratio (VBR), was comparable between controls and infected rats up to 30 hours post-infection at which time point 3 out of 4 infected rats had increased VBR compared to controls. When compared to controls, the VBR was significantly increased among infected rats in the total study period (6 to 48 hours, Two-way ANOVA, P < 0.0001) as well as the terminal phase of meningitis (36 to 48 hours, P < 0.0001), but not in the developing phase (6 to 30 hours, P = 0.40).

In the terminal disease phase, a significant association was found between VBR, severity of clinical disease and motor disability (Spearmann rank, rho = 0.62, P = 0.024 and rho = 0.57, P = 0.04) whereas an inverse correlation was found between increased VBR and the fraction of enhancing brain cortex indicative of increased BBB permeability (rho = -0.73, P = 0.01) as well as the muscle perfusion ratio (rho = -0.75, P = 0.019).

#### Loss of BBB integrity (Fig. 3f)

A measure of BBB integrity was obtained in 26 infected and 13 control rats. Increased BBB permeability, measured as the fraction of cortex voxels enhancing due to gadolinium leakage, was observed in one rat with meningitis as early as 6 hours after infection. From 36 hours onwards, all infected rats (n = 11) had increased BBB permeability when compared to corresponding controls (n = 5). The maximum fraction of enhancing voxels was found from 30 to 42 hours after infection. Compared to the control group, the total fraction of enhancing brain voxels was significantly increased during the full course of disease as well as in the terminal phase (6 to 48 hours, Two-way ANOVA, P = 0.0019 and 36 to 48 hours, P = 0.019), but did not quite reach significance during the developing phase (6 to 30 hours, P = 0.06).

#### Cerebral cortex ΔSI_(bolus) _values (Fig. 3j,3k and 3l)

ΔSI_(bolus) _values were obtained from 26 infected and 13 control rats. The marked shift towards enhancing cortex voxels, and thus increased BBB permeability, in rats with meningitis, was also apparent in the analysis of cortex ΔSI_(bolus) _values based on gadolinium bolus passage. In infected rats, the selected cortex region (Fig. 2) shifted from non-enhancing high-, medium- and low-ΔSI_(bolus) _values towards enhancing high-, medium-, and low-ΔSI_(bolus) _cortical values. This shift towards increased BBB permeability was significant in all 3 classes of ΔSI_(bolus) _values in comparison with the control groups (6 to 48 hours, Two-way ANOVA, high, P = 0.017, medium, P = 0.0018 and low, P = 0.012). However, the total fraction of high-, medium- and low-ΔSI_(bolus) _cortical values (non-enhancing + enhancing) did not change significantly among infected rats in comparison with controls in either the developmental stage (6 to 30 hours, Two-way ANOVA, high, P = 0.97, medium, P = 0.99, and low, P = 0.98), terminal stage (36 to 48 hours, high, P = 0.78, medium, P = 0.71, and low, P = 0.2) or full course of disease (6 to 48 hours, high, P = 0.8, medium, P = 0.76, and low, P = 0.9). Within the control group, one 24 hour and one 42 hour rat had markedly reduced ΔSI_(bolus) _values comparable to the most affected infected rats. The number of high ΔSI_(bolus) _values in muscle were equally low in these control rats.

#### Muscle ΔSI_(bolus) _values (Fig. 3i)

Data from 24/29 infected rats was successfully analyzed. The number of voxels representing high ΔSI_(bolus) _muscle values in infected rats, relative to corresponding controls at each time point, showed a steady decline as infection progressed (Linear regression analysis, borderline significant, P = 0.05).

#### Apparent Diffusion Coefficient in cerebral cortex and basal ganglia (Fig. 3g and 3h)

Apparent Diffusion Coefficient (ADC) values were obtained in 28 infected rats and 13 controls. Increased variability in ADC values in cerebral cortex and basal ganglia was observed among infected rats in comparison with the highly uniform control group.

However, no significant differences in cortex ADC was found comparing infected rats to controls in either developing or terminal disease stages (6 to 48 hours, Two-way ANOVA, P = 0.47; 6 to 30 hours, P = 0.12; 36 to 48 hours, P = 0.67). Also, basal ganglia ADC was not significantly different between infected rats and controls (6 to 48 hours, P = 0.38; 6 to 30 hours, P = 0.062; 36 to 48 hours, P = 0.47).

### Brain injury (Fig. 5)

**Figure 5:**
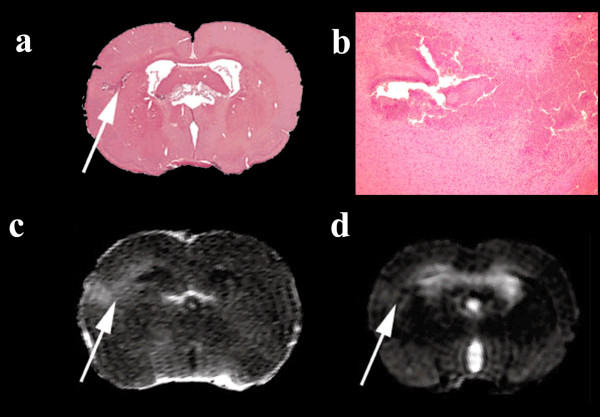
**Infection induced brain injury**. Example of an infected rat (48 hours) with a cortical abscess (white arrow), confirmed histopathologically (a and b), apparent in post-contrast T1W- and T2W images (c and d). The damaged area identified with histology covered only 1/3 of the area with contrast enhancement.

Focal injury to the brain parenchyma, other than hydrocephalus, was observed in four rats in the terminal stage of meningitis (two rats after 36 hours, one rat after 42 hours and one rat after 48 hours). Injury presented as a localized infarction, haemorrhage and abscess formation, the latter being readily observable on T1W post contrast images whereas the other lesions were smaller and less obvious on MR images.

## Discussion

Few studies have combined MRI and experimental pneumococcal meningitis research [10]. The present study is the first to use minimally invasive *in-vivo *MRI methods to describe the development of brain pathoanatomy and pathophysiology in a meningitis model. Within 24 hours after infection, MRI was able to detect physiological changes as the disease developed. In the terminal disease phase, the most marked results obtained with MRI were the significant expansion of ventricles and increased BBB permeability. Cortex brain water distribution (ADC) also changed but appeared to be subject to variation making interpretation of the data less obvious. Regions of high ΔSI_(bolus) _muscle values declined as infection progressed, whereas the included classes of cortex ΔSI_(bolus) _values were not subject to significant changes.

Hydrocephalus is a well-known complication of bacterial meningitis associated with poor outcome and brain injury [18-20], and preliminary studies directed at reducing intracranial pressure appear promising [21]. This study describes a marked expansion of ventricles that appears to develop, in the terminal disease stage [10] in close association with development of severe clinical disease and deteriorating motor performance. This is in agreement with recent findings of a close association between clinical disease score and intracranial pressure [22].

A hallmark of bacterial meningitis is the loss of BBB integrity allowing CSF leukocyte accumulation and diffusion of water and plasma constituents into brain parenchyma. In the present study, permeability of the vascular barrier increased markedly as meningitis progressed. This was visualized by the accumulation of contrast agent surrounding brain cortex and in the ventricles (Fig. 4). The loss of BBB integrity appeared to be an efficient disease biomarker, making it possible to identify infected and control animals at early time points after infection. In comparison to methodology previously applied to investigate BBB integrity such as Evans Blue staining [23], MRI enabled quantification and visualization of areas with increased BBB permeability that were confined to the outer layers of cerebral cortex (see Fig. 2). The close association between increased VBR and low fraction of enhancing cortex and thus a low BBB permeability suggests that expansion of the ventricles may influence cortex perfusion and via physical compression alter, for example, interstitial volume. This may account for the observed changes in brain water distribution and contrast agent kinetics even though the present study failed to identify the association between the two parameters.

Brain water content has in previous experimental meningitis studies been shown to increase as a consequence of the infectious and inflammatory response [24-26]. To some extent, this is in accord with our measurements of ADC, reflecting altered water distribution in the extracellular compartment (vasogenic oedema [27]) in cerebral cortex and basal ganglia in the majority of infected rats in the developmental stage of meningitis. Normalisation, or decrease, of ADC was observed in the terminal stage of meningitis and could be related to the concomitant ventricle expansion, as has previously been shown [28]. In the early stages of meningitis, increased brain water content may reflect increased blood volume. This would be in agreement with previous findings of increased cerebral blood flow in early meningitis and changes in ADC could thus be due to alterations in blood flow and volume [24,29]. This study has not investigated the distribution of water between tissue and ventricles. Future studies measuring ventricular size and ADC in combination with wet/dry weight analysis would provide further mechanistic insight into this characteristic of the disease.

Our measurement of ΔSI_(bolus) _values due to contrast agent induced T2* effects during the bolus passage in cerebral cortex was not subject to any significant overall changes whereas the area of high ΔSI_(bolus) _muscle values declined as infection progressed. The latter being in agreement with studies of muscle perfusion in septicaemia showing areas of well perfused muscle to decrease as septicaemia worsens [30-32]. Previous studies have shown that cerebral perfusion is compromised in bacterial meningitis as a consequence of systemic disease, brain oedema, and raised intracranial pressure [3,33,34]. Our experimental data cannot support this as a general assumption, since we found a large variation within ΔSI_(bolus) _categories in the terminal phase of meningitis where only seven of 15 rats between 30 and 48 hours after infection presented an increased fraction of low ΔSI_(bolus) _brain areas (Fig. 3l). Our ΔSI_(bolus) _results were limited to the cortex, a region that has previously been shown experimentally to be more well perfused, even in severely ill subjects [26], and in patients to be subject to great variation and regional differences [35]. It is important to state that the ΔSI_(bolus) _measure of 'perfusion' reported here is dependent on a number of other physiological factors and only partly on perfusion.

Several limitations in the presented study should be considered. Firstly, biological variation in disease development and the limited numbers of animals used makes temporal comparison sub-optimal. Whilst the sacrifice of rats at designated time points was performed to ensure histopathological evaluation, this limited the study of pathophysiological events preceding a poor outcome. Imaging of rats on more than one occasion would have significantly improved analysis of inter-relationships. Furthermore, the study design is limited by the relatively short time course of the disease and image acquisition times. The dynamic MRI measurements provided only a semi-quantitative measure that only partly reflects perfusion. Hence the crude categorization and dependency of the ΔSI_(bolus) _values on other parameters including blood volume clearly indicates that future studies could be further optimized. Quantitative perfusion measurements could be performed following sequence optimisation to acquire kinetic changes in relaxation times following gadolinium administration. The gadolinium concentration could then be fitted to appropriate kinetic models to obtain measurements of vascular permeability, extracellular volume and perfusion. Also, ADC measurements may be improved by introducing measurement of water motion in three or even more planes as compared to the single plane performed here. In addition, the necessary use of a total anaesthesia during scanning sessions affects pathophysiological parameters and result in changes in vasodilatation, blood pressure, blood supply or ventilation [36] in a disease-dependent way.

Even though our study was performed using a dedicated 4.7 Tesla animal scanner, the resolution was not optimal and small lesions in the cerebral cortex were not readily recognized as areas with lesions until after the histopathological evaluation of the corresponding specimen. The discrepancy between the extent of gadolinium distribution and histopathology shown in Fig. 5 is probably due to standard histological methods being unable to identify early neuronal loss since we have previously shown abscesses to be surrounded by a bright halo of ballooning or necrotic neurons [4]. The advantages of working at higher magnetic field strengths enabling higher resolution MRI images to be acquired and would be of significant benefit when comparing MR and histological images.

## Conclusion

The present study has shown the ability of MR methods to monitor *in-vivo *changes in brain pathoanatomy and pathophysiology as meningitis progresses. Measurement of BBB breakdown and dilation of ventricles has identified parameters that are related to disease severity. MRI is therefore of significant value for the development and evaluation of new and successful therapeutic approaches.

## Competing interests

The author(s) declare that they have no competing interests.

## Authors' contributions

CTB and IJR conceived the study, study design and coordination. CTB, IJR, HS and LVS participated in the experimental work. CTB, IJR, CØ, NFM and JDL performed data analysis and data interpretation. CTB and HS made ROI drawings. ML analyzed the dynamic data whilst LVS participated in the analysis of quantitative diffusion data.

CTB and IJR wrote the manuscript. All authors read and approved the final manuscript.

## Pre-publication history

The pre-publication history for this paper can be accessed here:


